# Retraction Note: Fabrication of a magnetic alginate-silk fibroin hydrogel, containing halloysite nanotubes as a novel nanocomposite for biological and hyperthermia applications

**DOI:** 10.1038/s41598-024-84517-7

**Published:** 2025-01-06

**Authors:** Reza Eivazzadeh-Keihan, Zahra Sadat, Hooman Aghamirza Moghim Aliabadi, Fatemeh Ganjali, Amir Kashtiaray, Milad Salimi Bani, Samira Komijani, Mohammad Mahdi Ahadian, Nabi salehpour, Reza Ahangari Cohan, Ali Maleki

**Affiliations:** 1 Nanobiotechnology Department, New Technologies Research Group, Pasteur Institute of Iran, Tehran, Iran; 2 Catalysts and Organic Synthesis Research Laboratory, Department of Chemistry, Iran University of Science and Technology, Tehran, 16846-13114 Iran; 3 Advanced Chemical Studies Lab, Department of Chemistry, K. N. Toosi University of Technology, Tehran, Iran; 4 Department of Biomedical Engineering, Faculty of Engineering, University of Isfahan, Isfahan, Iran; 5 Biotechnology Research Center, Pasteur Institute of Iran, No.358, 12 Farvardin St., Tehran, 1316943551 Iran; 6 Department of Biotechnology School of Biology, Alzahra University, Tehran, Iran; 7 Institute for Nanoscience and Nanotechnology, Sharif University of Technology, Tehran, Iran; 8 Department of Medical Biotechnology, Pasteur Institute of Iran, Tehran, Iran

Retraction of: *Scientific Reports* 10.1038/s41598-022-19511-y, published online 14 September 2022

Editors have retracted this Article.

After publication of this Article, concerns about the data were brought to the attention of the Editors. Specifically, the well images presented in Figures 4 and 5 appear to have been published in an article with common authors^1^ that was under review simultaneously to this article, where they are presented differently.

The Editors requested that the Authors provide full raw data and copies of their ethics approval and protocol for the use of blood samples drawn from a human donor, but the Authors were not able to do so. The Editors no longer have confidence in the reliability of the results and findings presented in this Article.

Reza Eivazzadeh-Keihan, Zahra Sadat, Hooman Aghamirza Moghim Aliabadi, Fatemeh Ganjali, Amir Kashtiaray, Milad Salimi Bani, Samira Komijani, Reza Ahangari Cohan and Ali Maleki disagree with this retraction. Mohammad Mahdi Ahadian and Nabi Salehpour did not respond to correspondence from the Editors regarding this retraction.
